# Relationship between Chinese college students’ attitude to physical exercise and psychological capital: the mediating effects of self-control and gender

**DOI:** 10.3389/fpubh.2024.1443489

**Published:** 2024-11-12

**Authors:** Xinnan Li, Lei Cui, Qi-Qi Shen, Rui Luo, Min Liu

**Affiliations:** ^1^College of P.E. and Sports, Beijing Normal University, Beijing, China; ^2^Department of Physical Education, Beijing Technology and Business University, Beijing, China; ^3^China University of Labor Relations, Beijing, China; ^4^College of Physical Education and Science, Qufu Normal University, Qufu, China

**Keywords:** attitude toward physical exercises, psychological capital, self-control, gender, college students

## Abstract

**Objective:**

This study aimed to explore the impact of attitudes toward physical exercise on college students’ psychological capital and its mechanism of action, providing new pathways and scientific evidence for enhancing psychological capital among college students.

**Methods:**

A total of 519 college students (mean age: 19.95 ± 1.34) were surveyed using a questionnaire.

**Results:**

(1) Attitudes toward physical exercise have a significant direct predictive effect on college students’ psychological capital; (2) Attitudes toward physical exercise can indirectly predict psychological capital through the mediating role of self-control; (3) The influence of attitudes toward physical exercise on self-control is moderated by gender, with a greater impact on female college students than on male students.

**Conclusion:**

(1) This study investigated the mechanism by which attitudes toward physical exercise influence psychological capital through self-control and gender differences; (2) The study revealed the positive role of psychological capital, with a significant predictive effect of attitudes toward physical exercise on college students’ psychological capital (*B* = 0.46, *p* < 0.001), and identified the reasons for gender differences, with attitudes toward physical exercise having a stronger promoting effect on self-control in female college students (*B* = 0.26, *t* = 3.80, *p* < 0.001). It provides guiding suggestions for enhancing psychological capital by fostering attitudes toward physical exercise among college students.

## 1 Introduction

In July 2021, the Ministry of Education issued a circular on strengthening the management of students’ mental health, stating: “Sports, aesthetic education, labour education, and campus culture play an important role in promoting the development of students’ mental health in all aspects.” On 29 November of the same year, the Ministry of Education convened a national meeting to promote the mental health education of university students. The meeting was deployed to promote the high-quality development of the mental health education of university students. Psychological capital is one of the important indicators that can reflect the positive psychological quality and physical and mental health development of college students. Having positive psychological capital is associated with increased self-confidence, perseverance, and an optimistic work ethic among college students to achieve positive contributions to their development (1). Studies have shown that attitudes toward physical activity as a psychological tendency influence college students’ physical activity (2), while physical activity can contribute to the overall physical and mental development of college students (3). In addition, for college students, promoting an active attitude toward physical exercise is one of the important ways to promote their overall physical and mental development (4). Therefore, it is worthwhile for physical education educators to explore whether the “physical exercise attitude” with high intervention efficiency and low intervention cost can promote psychological capital, so as to give full play to the role of physical education in improving the mental health of college students. This study intends to investigate the relationship between the attitude toward physical exercise and psychological capital and explore the potential mediating and regulating variables of the relationship between the attitude toward physical exercise and psychological capital of college students, so as to provide empirical evidence for the development of economic and effective sports intervention programs to promote the psychological capital of college students.

In the current educational context, the Ministry of Education has explicitly stated that physical activities play a crucial role in promoting the comprehensive development of students’ psychological health. This study responds to this policy direction by focusing on the relationship between attitudes toward physical activity and college students’ psychological capital for the first time, thereby filling a gap in existing research regarding the application of physical activities in the field of mental health education. The novelty of this study lies in its exploration of the direct impact of attitudes toward physical activity on psychological capital, as well as an in-depth analysis of potential mediating and moderating mechanisms, providing a new theoretical perspective on how physical activities can promote the psychological health of college students. Furthermore, this study takes into account the economic viability and efficiency of intervention measures, offering empirical evidence for the development and implementation of cost-effective physical activity intervention programs. Through these research contributions, this study aims to provide strategic recommendations for physical education professionals and mental health practitioners to foster positive psychological development and overall wellbeing among college students.

### 1.1 Psychological capital

Psychological capital is one of the important indicators of positive psychological quality of college students. It is the positive psychological ability that college students possess during their growth and development. Its components include self-efficacy, hope, optimism, and resilience (5). Among them, self-efficacy refers to the confidence and ability to make the necessary efforts to succeed in the face of challenging work. Hope means persevering with the goal and adjusting the path to the goal if necessary for success; Optimism refers to a positive attribution of present and future success; and Resilience is the ability to persevere, to overcome external and internal difficulties, to recover quickly, and to persevere in the tasks undertaken, even in adversity and adverse circumstances (5).

Psychological capital is a psychological resource that lies between state and idiosyncrasy. It is an important factor in an individual’s ability to gain a competitive advantage and engage in positive behavior (6). It is a renewable, untapped, and sustainable competitive resource for talent (7). Psychological capital plays an important role in college students’ study, life, and work. Studies have shown that positive mental capital helps college students develop positive attitudes and healthy behaviors, improves academic performance, mental health, and life satisfaction (8, 9), and regulates anxiety and depression levels (10).

### 1.2 Relationship between physical exercise attitudes and psychological capital

Physical activity attitude refers to the comprehensive expression of an individual’s attitude toward physical education and physical activity (64), including the three elements of an individual’s own cognitive evaluation of physical education, emotional experience, and behavioral intention. Emotional experience refers to an individual’s interest, confidence, and emotional factors experienced and stimulated when participating in physical exercise. Behavioral intention refers to whether an individual has the intention of participating in exercise, can form a certain persistence, and produce a good exercise effect (11, 12). Studies have shown that physical activity has a significant positive effect on many dimensions of college students’ mental capital (13–18). As far as self-efficacy is concerned, physical exercise can help college students improve their mental toughness and happiness and keep them positive and optimistic, which in turn improves their sense of self-efficacy (19, 20). In addition, intervention studies have shown that moderate intensity of physical activity can effectively boost both the optimism and resilience dimensions of mental capital (19). This may be because physical activity allows exercisers to increase their sense of self-efficacy by mastering a variety of motor skills and successful experiences, leading to a more competitive mental capital, especially for their level of optimism and resilience development (21). The Theory of Planned Behavior (TPB) is a well-known theory in the field of exercise behavior modification, which assumes that behavior is determined by intention and that an individual’s attitude toward behavior is an integral part of that intention (22). Therefore, as an important factor influencing the physical activity behavior of college students (2, 23, 37), physical activity attitude may promote the formation of positive psychological capital. In light of the foregoing, we propose the following assumptions:

*Hypothesis 1*: Physical activity attitudes can positively predict college students' psychological capital.

### 1.3 Intermediating self-control

Self-control refers to a person’s tendency to overcome innate desires, habits, or innate behavioral responses, as well as the ability to sustain adaptive behavior consistently. Its role is to improve people’s ability to resist short-term temptations, adhere to social rules and norms, establish good behavior habits, and develop self-restraint in order to achieve long-term goals (24).

Physical activity attitudes may predict higher levels of self-control. Empirical research shows that physical activity attitude is closely related to physical activity behavior (2), and physical activity behavior is an effective way to improve college students’ self-control ability (25, 26). In complex physical training situations, individuals need to adjust and change strategies in time to the complex situations of peer and opponent behavior and on-court situations, using cognitive control functions dominated by the more complex frontal lobes of the brain. Thus, long-term exercise of this kind improves self-control (27). Self-control energy models suggest that the implementation of any self-control process requires the consumption of a holistic, short-term cognitive control resource of limited content (28), which, like the body’s muscle power, decreases when the self-control resource is depleted. Self-control energy can also be restored through rest (29) and improved through training (30). In physical exercise, increasing intensity and load while depleting self-control resources can lead to a physiological “pole.” By persisting and pushing the limits, an individual can improve their self-control ability (25).

In the relationship between self-control and psychological capital, empirical studies have shown that self-control can promote the formation and development of psychological capital in college students (31, 32) and increase the number of resources of psychological capital in college students (33, 34). Empirical studies have shown that individuals with strong self-control are able to engage in positive mental regulation and are more likely to exhibit positive emotions such as happiness, focus, and pride, resulting in greater mental toughness and a higher sense of self-efficacy (35, 36, 63). Additionally, levels of self-control can positively predict mental health (63). Psychological capital is an important indicator of positive psychological quality and the physical and mental health development of college students (1). Therefore, the success or failure of self-control may have a great impact on the psychological capital of college students. In conclusion, the study makes the following assumptions:

*Hypothesis 2*: Self-control acts as an intermediary in the relationship between physical exercise attitudes and psychological capital. Specifically, the attitude of physical exercise can improve self-control, and self-control can positively predict positive psychological capital.

### 1.4 Regulating effects of gender

Gender is an important variable in examining the mechanism of how college students’ attitudes toward physical exercise impact their psychological capital. The study found that male college students were more physically active and motivated overall than their female counterparts (37, 38), and male students were more likely to participate more frequently (23, 38). However, under conditions of frequent exercise, we found that will occur, the practice or the habit effect that causes physical exercise to the male university student self-control promotion is relatively weak or not obvious. In other words, the attitude of physical exercise may have a more positive effect on female college students’ self-control than that of male college students. Therefore, this study assumes the following:

*Hypothesis 3*: The relationship between physical activity attitudes and self-control is regulated by gender. Specifically, there was a stronger relationship between physical activity attitudes and self-control among female college students.

**Figure 1:**
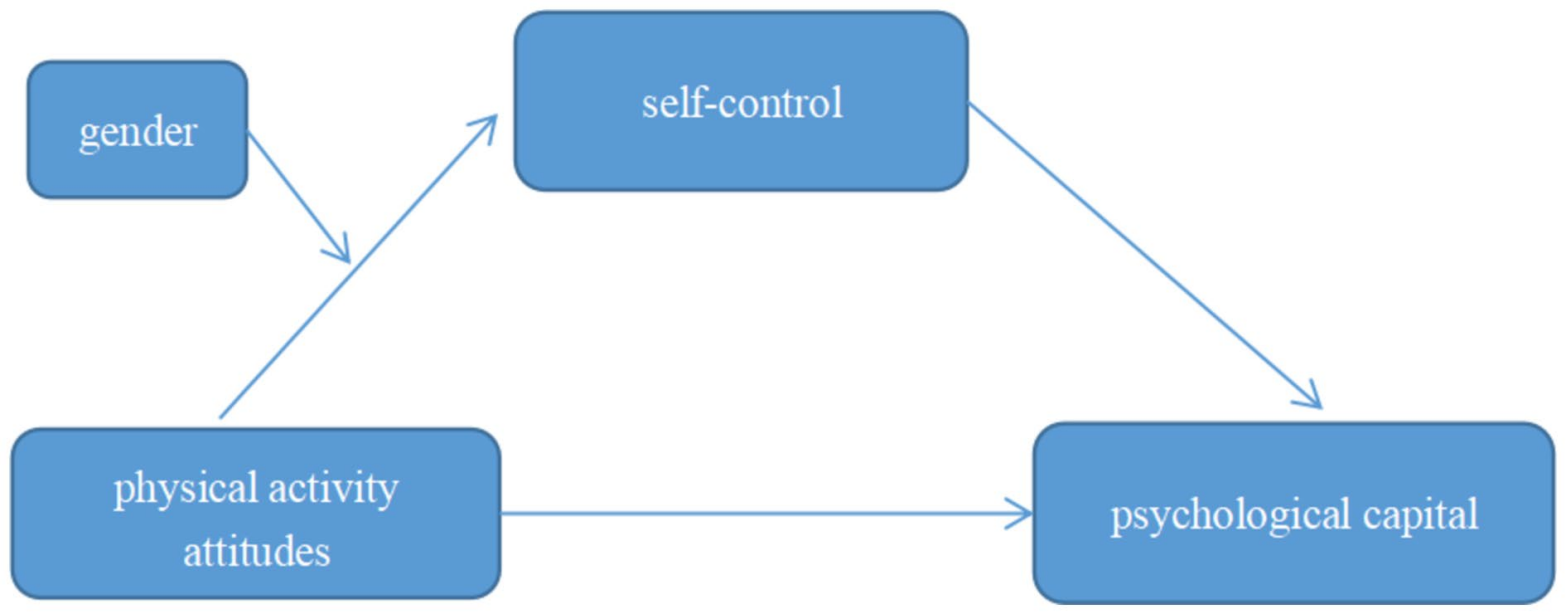
Research hypothesis model.

Based on this hypothesis, this study focuses on university students to explore this question further and constructs a regulated intermediary model (Figure 1).

### 1.5 Research purpose

The primary purpose of this study is to investigate the relationship between college students’ attitudes toward physical exercise and their psychological capital and to explore the underlying mechanisms and moderating effects that may influence this relationship. Specifically, the study aims to examine the direct effect of attitudes toward physical exercise on the psychological capital of college students; identify the mediating role of self-control in the relationship between attitudes toward physical exercise and psychological capital; investigate the moderating effect of gender on the relationship between attitudes toward physical exercise and self-control, and consequently on psychological capital; and provide empirical evidence for the development of cost-effective and efficient exercise intervention programs aimed at enhancing psychological capital among college students.

By achieving these objectives, the study intends to contribute to the body of knowledge on the role of physical education in promoting mental health and wellbeing among college students. Furthermore, the findings are expected to offer practical implications for educational institutions and policymakers to design and implement strategies that foster a positive attitude toward physical exercise, thereby enhancing the psychological capital of the student population.

## 2 Methods

### 2.1 The participants

A total of 600 college students [age (19.95 ± 1.34) years] were selected as research subjects using a cluster sampling method. After excluding 81 incomplete or invalid questionnaires due to inattentive responses, 519 valid questionnaires were retained, resulting in an effective rate of 86.5%. Among them, there were 232 male college students (44.7%) and 287 female college students (55.3%). Among the participants, approximately 20% (104 individuals) reported having experience as student leaders, with roles ranging from class monitors and league branch secretaries to student council presidents, indicating involvement in student organizations at various levels.

To assess the economic status of the participants’ families, a classification based on annual household income was established, dividing the sample into three categories: low (with an annual income below 40,000 RMB, accounting for 30% of the sample, *n* = 156), medium (with an annual income between 40,000 and 80,000 RMB, accounting for 40% of the sample, *n* = 208), and high (with an annual income exceeding 80,000 RMB, also accounting for 30% of the sample, *n* = 156). This classification facilitates the examination of how different economic backgrounds may influence students’ attitudes toward physical exercise and their psychological capital.

### 2.2 Research tools

#### 2.2.1 Physical activity attitude scale

The scale uses the Physical Exercise Attitude Scale compiled by Mao Rongjian (12) to measure college students’ exercise attitude levels. The research scale consists of 70 items and consists of eight dimensions: behavioral attitudes, target attitudes, behavioral cognition, behavioral habits, behavioral intentions, emotional experiences, behavioral control, and subjective criteria. Using the 5-point Likert scoring method, from “1” to “5” is completely inconsistent. The higher the participants’ overall score, the higher the participants’ attitudes toward exercise. The scale demonstrated a good fit in this study, with χ^2^/df = 3.45, RMSEA = 0.059, CFI = 0.99, and TLI = 0.98. Cronbach’s *α* coefficient for the scale in this study was 0.97.

#### 2.2.2 Mental Capital Scale (PPQ)

The scale is based on the Positive Psychological Capital Questionnaire developed by Zhang Kuo (39), which has a total of 26 questions divided into four dimensions: self-efficacy, optimism, hope, and resilience. The 7-point Likert scoring method was used in the questionnaire, with a score of 1 for “complete non-conformity” and 7 for “complete conformity,” with progression in the middle. The higher the participants’ scores, the higher their overall level of psychological capital. The scale demonstrated good validity and model fit, with χ^2^/df = 0.17, RMSEA = 0.07, CFI = 0.97, and TLI = 0.95. Cronbach’s *α* coefficient for the scale in this study was 0.93.

#### 2.2.3 Self-Control Scale (SCS)

In this study, the self-control scale, as revised by Shuhua Tan and Yongyu Kuo (40), was used to measure individual self-control. The scale has 19 items, including five dimensions: impulse control, healthy habits, resistance to temptation, focus on work, and abstinence from entertainment. The questionnaire uses a 5-point scale, with one meaning “completely inconsistent” and five meaning “fully compliant.” The higher the participants’ total score, the higher their level of self-control. The scale demonstrated good validity and model fit with χ^2^/df = 1.533, RMSEA = 0.05, CFI = 0.93, and TLI = 0.93. In this study, the total scale’s Cronbach’s *α* coefficient was 0.89.

### 2.3 Research design

This study aims to systematically explore the relationship between college students’ attitudes toward physical exercise and psychological capital, employing the following research design strategies: (1) Sampling Method: Cluster sampling was utilized to ensure a representative sample from various colleges and grades of college students. (2) Test Administrator Training: All physical education teachers responsible for the testing underwent standardized training to ensure consistency and accuracy in the testing process. (3) Informed Consent: Prior to testing, participants were clearly informed about the purpose, procedures, and confidentiality principles of the study to ensure voluntary participation based on complete information. (4) Anonymous Questionnaire Survey: The questionnaire was conducted anonymously to reduce the impact of social expectations on responses and was collected immediately after completion to ensure data integrity. (5) Testing Environment Control: The test was conducted in a standardized physical education class environment, providing quiet and undisturbed testing conditions for each participant. (6) Time Efficiency: The questionnaire was designed with an emphasis on efficiency, with an average completion time controlled within 20 min to minimize disruption to participants’ normal study activities.

### 2.4 Statistical methodology

This study utilized SPSS 25.0 and the PROCESS 4.0 plugin for data management and analysis.

This study employs a suite of statistical methods to ensure the rigor of data analysis. Common method bias is initially assessed using the Harman single-factor model and further substantiated through principal component analysis, ensuring the reliability of the results. Descriptive statistics, including means and standard deviations, provide a fundamental description of the data characteristics for each variable. The interrelationships among variables are quantified using Pearson product–moment correlation coefficients, revealing the associations between attitudes toward physical exercise, self-control, and psychological capital. Mediation effect analysis is conducted utilizing the non-parametric percentile bootstrap method available in the SPSS PROCESS macro, which enhances the robustness of the estimation through 5,000 resampling iterations. Furthermore, this study takes into account potential moderating variables, such as gender, by constructing moderated mediation models to evaluate their impact on the primary relationships. All analyses are conducted while controlling for student leadership experience and family economic status to mitigate potential confounding effects. Statistical significance is denoted by asterisks to distinguish different levels of significance (*p < 0.05, **p < 0.01, ***p < 0.001), ensuring the clear communication of results.

## 3 The results

### 3.1 Common method bias control and testing

Anonymous method and reverse scoring of some entries were used to control the measurement in this study. Common methodological deviations after data collection were examined using the Harman single-factor model method (41). In this assay, unrotated principal component analysis was performed simultaneously for all variable items and revealed that there were 23 factors with an eigenvalue greater than 1, with the first factor extracted explaining 25.50% of the total variation, below the threshold of 40%, suggesting that common method bias did not significantly impact the results.

### 3.2 Mean values, standard deviations, and correlation matrices of the study variables

Table 1 presents the means, standard deviations, and Pearson correlation matrix for each variable. The results indicate that there are significant positive correlations between attitudes toward physical exercise and self-control (*r* = 0.411, *p* < 0.01), as well as between attitudes toward physical exercise and positive psychological capital (*r* = 0.495, *p* < 0.01). This suggests that individuals with more positive attitudes toward physical exercise are likely to exhibit better self-control and possess higher levels of psychological capital. Self-control is also significantly and positively correlated with positive psychological capital (*r* = 0.589, *p* < 0.01), which may reflect the advantage of individuals with stronger self-control in building psychological capital.

**Table 1:** Mean values, standard deviation, and correlation matrices of the variables studied (*n* = 519).

	1	2	3	4	5
1. Attitude toward physical exercise	1				
2. Self-control	0.411**	1			
3. Positive psychological capital	0.495**	0.589**	1		
4. Student cadre experience	0.088*	0.007	0.142**	1	
5. Family economic status	0.152**	0.169**	0.264**	0.159**	1
M	3.32	3.24	4.80	0.50	1.55
SD	0.535	0.613	0.823	0.500	0.543

*denotes *p* < 0.05, **denotes *p* < 0.01, **denotes the same under *p* < 0.001.

Student leadership experience shows a low but significant positive correlation with attitudes toward physical exercise (*r* = 0.088, *p* < 0.05) and positive psychological capital (*r* = 0.142, *p* < 0.01), which may imply that the role of being a student leader is associated with a more positive attitude toward physical exercise and higher levels of psychological capital. However, the strength of these relationships is relatively low, indicating that other factors may play a more significant role in the relationships among these variables.

Family economic status is significantly and positively correlated with attitudes toward physical exercise (*r* = 0.152, *p* < 0.01), self-control (*r* = 0.169, *p* < 0.01), and positive psychological capital (*r* = 0.264, *p* < 0.001), indicating that students from better-off families may score higher in these aspects.

The standard deviation results suggest that family economic status (SD = 0.543) has the greatest variability, while student leadership experience (SD = 0.500) has relatively less variability, which may reflect a broader diversity in family economic status and a more concentrated distribution of student leadership experience in the sample.

Additionally, research has shown that there are significant differences in psychological capital in terms of gender, age, major, and student leadership experience, with men generally scoring higher in psychological capital than women (42); psychological capital increases with age (43, 44); and individuals with higher education have higher levels of optimism and self-confidence factors in psychological capital (45). Among the factors of psychological capital, students majoring in science and engineering score higher in resilience than those majoring in liberal arts (46). There are significant differences in the four dimensions of psychological capital in terms of student leadership experience and grade level (47). Therefore, this study also examined the relationship between positive psychological capital and individual background information such as age, major, student leadership experience, and family economic status, finding that positive psychological capital is also related to student leadership experience and family economic status, with specific results shown in Table 1.

### 3.3 Self-control intermediation testing

Intermediate effect testing was conducted using the non-parametric percentile bootstrap method with bias correction, as implemented in SPSS PROCESS 4.0 (48). Adopting bias-corrected percentile bootstrap with 5000 replications for mediation effect analysis. According to the results of the correlation analysis in 3.2, there is a relationship between students’ cadre experience, family economic situation, and psychological capital. The present study controlled for these variables during the data analysis.

The results showed that after controlling for student cadre experience and family economic status, the direct predictive effect of physical activity attitude on mental capital was significant (*B* = 0.71, *p* < 0.001). In the mediating effect model, the direct predictive effect of attitudes toward physical activity on mental capital was significant (*B* = 0.43, *p* < 0.001). Additionally, the positive predictive effect of physical activity attitude on mental capital (*B* = 0.46, *p* < 0.001), and the indirect effect of physical activity attitude on mental capital was significant (*B* = 0.60, *p* < 0.001). The direct effect of attitudes toward physical exercise on psychological capital, along with the mediating effect of self-control, shows Bootstrap 95% confidence intervals that do not include zero (as shown in Table 2). This indicates that attitudes toward physical exercise can not only directly affect psychological capital but also indirectly affect it through self-control. The direct effect (0.43) and mediating effect (0.28) account for 60.05 and 39.44%, respectively, of the total effect (0.71). Details are presented in Table 2.

**Table 2:** A 95% confidence interval of non-parametric percentile bootstrap for effect decomposition and bias correction.

Pathway	Effect value	*p*	Bootstrap standard error	Confidence interval	
				Lower bound	Upper bound
Physical Exercise Attitude → Psychological Capital	0.43	<0.001	0.06	0.33	0.54
Physical Exercise Attitude → Self-Control → Psychological Capital	0.28	–	0.04	0.20	0.36
Total effect	0.71	<0.001	0.06	0.60	0.82

### 3.4 Relationship between physical activity attitudes and psychological capital: adjusted intermediate model testing

According to Wen Zhonglin and Ye Baojuan (49), all predictive variables were standardized in each equation, while student cadre experience and family economic status were controlled. The parameters of the three regression models are estimated as follows: Model 1 examined the regulatory effect of gender on the relationship between physical activity attitudes and psychological capital; Model 2 examined the regulation effect of gender on the relationship between physical activity attitude and self-control. Model 3 examined the predictive effect of physical activity attitude, self-control, and gender interaction on psychological capital.

As shown in Table 3, in model 1, physical activity attitudes significantly positively predicted mental capital (*B* = 0.46, *p* < 0.001), but the interaction of sexual activity and physical activity attitudes did not significantly predict mental capital (*B* = −0.01, *p* = 0.979). Physical activity attitudes positively predicted self-control (*B* = 0.39, *p* < 0.001), and the interaction of sexual activity and physical activity attitudes significantly predicted self-control (*B* = 0.08, *p* < 0.05). In Model 3, physical exercise attitudes positively predicted psychological capital (*B* = 0.28, *p* < 0.001). Additionally, self-control also significantly positively predicted psychological capital (*B* = 0.45, *p* < 0.001), but the interaction term of gender and self-control had no significant effect on the prediction of psychological capital (*B* = −0.02, *p* = 0.576).

**Table 3:** Regulating effect of gender on self-control mediating effect.

Predicted variables	Model 1: Mental capital			Model 2: Self-control			Model 3: Mental capital		
	*B*	*SE*	*t*	*B*	*SE*	*t*	*B*	*SE*	*t*
Physical activity attitudes	0.46***	0.04	13.05	0.39***	0.04	9.73	0.28***	0.04	7.92
Gender	0.06	0.04	1.57	0.02	0.04	0.38	0.05	0.03	1.55
Self-control							0.45***	0.04	12.45
Gender × physical activity attitudes	−0.001	0.04	−0.18	0.08*	0.04	1.97			
Gender × self-control							−0.02	0.03	−0.56
Family economic status	0.18***	0.04	4.66	0.11*	0.04	2.78	0.13***	0.03	3.72
Student cadre experience	0.07	0.04	1.82	−0.04	0.04	−1.01	0.09***	0.03	2.65
Adjusted *R*^2^	0.28			0.18			0.45		
*F*	42.00***			23.96***			71.37***		

Based on the above results, it can be found that four variables, namely physical activity attitude, self-control, psychological capital, and gender, constitute a mediated model in which gender regulates the impact of physical activity attitude on psychological capital (first half of the pathway).

**Figure 2:**
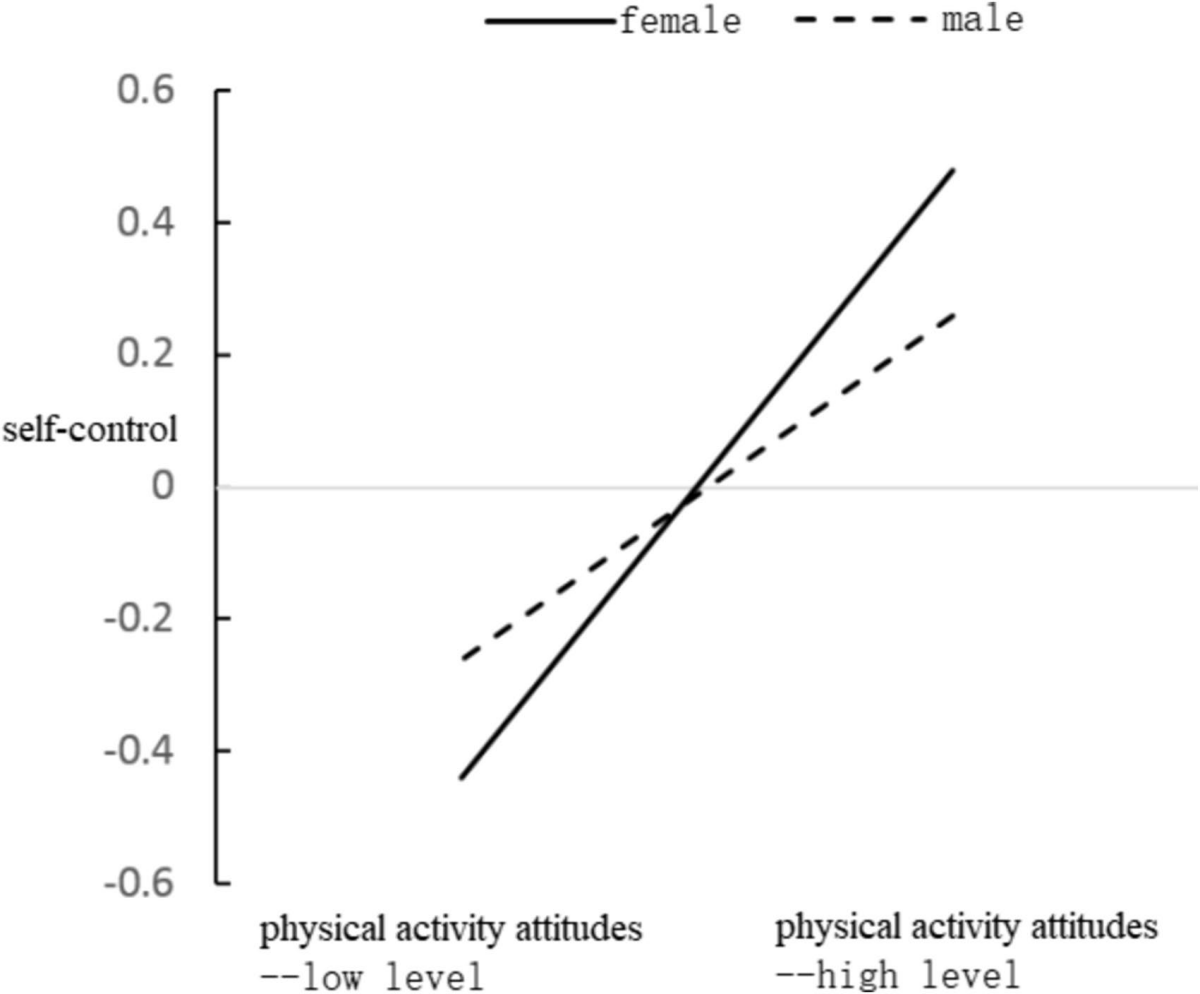
Gender role in regulating the relationship between physical activity attitude and self-control.

To better elucidate the moderating effect of gender, a simple effects analysis graph was drawn based on the different genders using the language of academic research in psychology. The results are shown in Figure 2. It can be seen that for female college students, their self-control level significantly increases with an increase in their sports attitude (*B* = 0.26, *t* = 3.80, *p* < 0.001), and the self-control level of male college students also significantly increases with an increase in their sports exercise attitude (*B* = 0.46, *t* = 9.16, *p* < 0.001). However, in comparison, the increase in self-control levels for female college students is greater. The moderation effect was tested using the PROCESS 4.0 plugin in SPSS (48), with 5,000 resamples. The results indicated that the 95% confidence interval of the moderation effect was [0.003, 0.163], which does not include 0, further demonstrating the significant moderating effect of gender. Relative to male college students, female college students exhibit a greater increase in self-control levels, with an increase in their sports exercise attitudes.

In general, the self-control mediating effect was greater in the group of female university students, as shown in Table 4.

**Table 4:** Intermediate effect of self-control in college students of different gender.

	Mediation effect size	Bootstrap standard error	95% confidence interval	
			Lower limit	Upper limit
Male college student	0.15	0.04	0.08	0.23
Female college student	0.21	0.04	0.15	0.28

## 4 Discussion

This study selected college students as the research object and constructed a regulated intermediary model. The results show that (1) the attitude of college students to physical training can positively predict the psychological capital of college students; (2) self-control acts as an intermediary in the relationship between physical exercise attitudes and psychological capital; and (3) gender plays a moderating role in attitudes to physical exercise and self-control. Compared to male college students, attitudes toward physical exercise have a greater impact on female college students. The results will be helpful in understanding the mechanism of positive psychological capital of college students and will have practical significance in improving their positive psychological quality and self-control ability.

### 4.1 Relationship between physical exercise attitudes and psychological capital

The results of this study show that there is a positive predictive relationship between college students’ attitudes toward physical exercise and psychological capital. Previous studies have mostly focused on the role of physical activity in promoting positive mental capital (15, 16, 18, 19). According to the theory of planned behavior, the attitudes that individuals develop after conceptualizing a particular event are important predictors of behavior (22). Therefore, college students with a positive attitude toward physical exercise tend to have higher executive power over physical exercise. Frequent physical exercise promotes exercisers to master various motor skills, which leads to successful experiences and feelings of self-efficacy. Experiences of overcoming difficulties and breaking new ground during exercise increase mental toughness, which is an important component of mental capital (21). Thus, attitudes (physical activity attitudes) drive behavior (regular exercise), and behavior influences cognition (positive mental cognition, such as mental capital), a pathway established in this study and supported by empirical evidence (50).

### 4.2 Intermediating self-control

This study also explores the mechanism of self-control in the relationship between physical exercise attitude and psychological capital. Intermediary variables can reveal the intrinsic mechanism of the influence of physical exercise attitude on psychological capital. The results show that the active attitude of physical training can improve the level of self-control, and the improvement of self-control can improve the positive psychological capital of college students (60).

In the relationship between physical activity attitudes and self-control, the results were consistent with previous studies that individuals with good physical activity attitudes improved self-control and individual toughness by engaging in more physical activities (51, 52). The results of this study are consistent with the Strength Model of Self-Control, which explores individual self-control based on “energy.” Self-control is seen as the body’s muscle, and constantly pushing boundaries can make muscles stronger. Similarly, self-control can be enhanced through exercise (24, 30, 53, 61).

In the relationship between self-control and psychological capital, the results of this study are consistent with previous studies that show that self-control predicts positive psychological capital (31–33). Dhar and Wertenbroch (54) argue that exercising self-control and resisting temptation can send clear positive self-signals. Guo Xiaoli et al. believe that controlling impulses and resisting temptations in life will lead to greater cognitive, emotional, and behavioral unity, less alienation or disobedience, and therefore a more positive mental state (55).

### 4.3 Regulating the effects of gender

Self-control acts as an intermediary between the attitudes of college students toward physical exercise and their psychological capital. The first pathway of gender regulation of this intermediate model is that there is a stronger relationship between the attitude toward physical exercise and self-control in female college students. This may be due to the fact that male students tend to be more motivated to exercise than female students (37, 38) and that they also exercise significantly more than female students (23, 38). As a result, there may be habit effects and potential bottlenecks in the benefits derived from physical activity. Therefore, the effect of physical exercise on the self-control of male college students may be weaker than that of female college students. In addition, the attitudes of male college students to physical exercise are more of a form of social entertainment; female college students’ attitudes toward physical exercise are mainly that they can maintain health and control their body shape. Compared to male college students, female college students pay more attention to external image and body image, therefore, female college students’ attitudes toward physical exercise are more related to body management. However, body management exercises require more self-control, which may explain why, among female college students, physical exercise attitudes have a significant impact on self-control abilities.

Gender role theory, which describes the behavioral systems that individuals acquire through cultural influences based on their gender, can help explain the findings of this study (56). From the viewpoint of the gender role theory, gender stereotyping of women in Chinese cultural background is mostly introverted, reserved, and restrained, so to some extent, female university students have more self-control ability (62). In this study, female college students with high self-control were more likely than male students to improve their self-control in their study lives, so the improvement in self-control was more pronounced among female college students.

### 4.4 Proposing new correlations

The findings of this study offer novel theoretical perspectives on the relationship between attitudes toward physical exercise and psychological capital at multiple levels. Initially, our results not only substantiate the existing theory that there is a positive association between attitudes toward physical exercise and psychological capital but also add new theoretical depth to this field by revealing the mediating role of self-control and the moderating role of gender (59).

Particularly, we found that the positive impact of attitudes toward physical exercise on self-control is greater in female college students than in males, a result that highlights the significance of gender in the formation process of psychological capital and contrasts sharply with previous studies that focused primarily on the overall effects of physical exercise behavior. Our findings further extend the theory by Babalola (45) regarding the impact of education level on psychological capital, suggesting that attitudes toward physical exercise may indirectly affect psychological capital by enhancing self-control capabilities.

Moreover, our study’s results resonate with the research of Megan (51) and Xie Jing (52), which emphasized the role of physical exercise behavior in improving self-control and resilience. However, our study goes further to point out that this impact varies by gender, possibly related to gender role expectations and socialization processes. This is indirectly supported by the research of Natalie et al. (21), who found that physical exercise can enhance an individual’s sense of self-efficacy and psychological resilience.

Our study’s results also complement the Theory of Planned Behavior (22) by proposing that attitudes toward physical exercise directly affect behavior and indirectly influence psychological capital through the mediating role of self-control. Finally, our findings provide new empirical support for the gender role theory (56), especially in terms of the relationship between attitudes toward physical exercise and self-control. Among female college students, the relationship between attitudes toward physical exercise and self-control is more pronounced, which may be related to the higher attention women pay to health and body image.

By proposing these new correlations, this study not only provides new insights into the mechanism of psychological capital formation among college students but also points the way for future research. Future studies can further explore how gender modulates the relationship between attitudes toward physical exercise and other psychological variables in different cultural contexts, and how these relationships affect the construction and development of psychological capital.

## 5 Limitations and future directions

The limitations of this study are as follows: First, due to the cross-sectional nature of the data, causation cannot be inferred. Future researchers will need to use longitudinal data to further confirm causality (e.g., to study whether the relationship between variables in the model is bidirectional). Second, all the scales used in this study are self-reported, which may be influenced by social approval bias. Future research should consider collecting objective data for a more in-depth analysis. Third, the participants in this study were Chinese college students and were in the early years of their freshman and sophomore years, so the results should be interpreted with caution when generalizing to other populations or age groups. Fourth, other demographic variables that may be related to psychological capital, such as academic background, being an only child, family status, and urban and rural household status, are not included in the statistical analysis. Future studies should include as comprehensive a control variable as possible to ensure scientific and generalizable results. Fifth, according to the Health Action Process Approach (HAPA), people’s healthy behavior changes are classified into stages of intention, planning, and action (57). Different regulatory variables, including self-efficacy, influence exercise behavior at various stages, creating a gap between intention and actual exercise, even with ongoing support for behavior change (58). Future research should examine the mediating and regulating variables between physical activity attitude/intention and physical activity from a social and psychological point of view, further consolidate the results of this study, and expand the programs and ideas of psychological capital intervention.

## 6 Conclusion and implications

### 6.1 Positive correlation between physical activity attitude and psychological capital

The research finds a significant positive correlation between physical activity attitudes and the psychological capital of college students. This finding underscores the critical role of physical activity attitudes in promoting the positive psychological development and overall wellbeing of college students. Physical educators should focus on cultivating positive attitudes toward physical exercise among students to stimulate intrinsic motivation, thereby enhancing the consciousness and sustainability of physical activity.

### 6.2 Mediating role of self-control

Self-control ability is key to an individual’s self-development and social adaptability. This study indicates that by enhancing college students’ positive attitudes toward physical training, their self-control ability can be effectively improved, which in turn enhances psychological capital. In physical education, students should be encouraged to set physical exercise plans and gradually increase the intensity of exercise to cultivate self-control and promote the enhancement of psychological capital.

### 6.3 Moderating effect of gender on the relationship between physical activity attitude and self-control

The study reveals the moderating effect of gender in the relationship between physical activity attitudes and self-control, especially among female college students, where strengthening physical activity attitudes has a more pronounced impact on improving self-control abilities. Therefore, physical educators should consider gender differences, pay attention to the differences in physical exercise attitudes between male and female college students, and provide additional training or guidance for male college students to enhance their self-control abilities. Guiding them to form a sense of purpose and meaning in physical exercise can then promote an improvement in their physical activity attitudes and self-control pathway.

In summary, this study highlights the importance of physical activity attitudes in promoting the development of college students’ psychological capital and reveals the key roles of self-control and gender. These conclusions provide new perspectives and strategies for physical education practice to more effectively promote the mental health and comprehensive development of college students.

## Data Availability

The raw data supporting the conclusions of this article will be made available by the authors, without undue reservation.
